# Discrimination of Tunisian Honey by Mineral and Trace Element Chemometrics Profiling

**DOI:** 10.3390/foods10040724

**Published:** 2021-03-29

**Authors:** Giuseppa Di Bella, Angela Giorgia Potortì, Asma Beltifa, Hedi Ben Mansour, Vincenzo Nava, Vincenzo Lo Turco

**Affiliations:** 1 BioMorf Department, University of Messina, Viale Annunziata, Polo Universitario, 98168 Messina, Italy; gdibella@unime.it (G.D.B.); vnava@unime.it (V.N.); vloturco@unime.it (V.L.T.); 2 APAE Higher Institute of Applied Sciences and Technology, University of Monastir, 5100 Mahdia, Tunisia; beltifa.asma@hotmail.com (A.B.); hdbenmansour@gmail.com (H.B.M.)

**Keywords:** honey, mineral elements, trace elements, ICP-MS, chemometric analysis, dietary exposure, Tunisia

## Abstract

The concentrations of 19 chemical elements have been determined in 36 honey samples of different botanical (wildflower, eucalyptus, eucalyptus red flowers, prickly pears, lemon blossom, thyme, almond, rosemary and jujube) honeys from the three geographical areas of Tunisia (Sidi Bouzid, Nabeul and Sfax) using inductively coupled plasma mass spectrometry (ICP-MS). The aim of this work was to use the multielement analysis together with chemometric tools to verify the botanical and the geographical origin of honeys. The correlation on the basis of mineral element content between the honey samples and their botanical and/or geographical origins was in some measure achieved. The data collected on the samples were also used to evaluate the nutritional quality and the potential health risks associated with elements via consumption of the Tunisian honey. According to the results obtained, the intake of essential elements was small, and the potential health risks associated with toxic or potentially toxic elements via consumption of this food were overall insignificant.

## 1. Introduction

Honey is one of the food commodities that is utilized worldwide as a direct food or as an ingredient in a range number of manufactured foods [1]. It is a concentrated aqueous solution of glucose and fructose that contains a mixture of other carbohydrates, proteins and amino acids, vitamins, enzymes, organic acids, lactones, minerals, colloids, aromatic substances, pigments, waxes and pollen that make it a very complex matrix [2,3,4,5,6,7]. Furthermore, numerous contaminants can be found in honey [8,9]. The mineral contribution accounts for 0.1–0.2%; it is constituted by 45–85% of K followed by Na, Ca and Mg. Honey contains also Cu, Fe, Zn and Mn in medium quantities and trace elements at much lower levels [10,11].

According to Council Directive 2001/110/EC of 20 December 2001 relating to honey, the country or countries of origin where the honey has been harvested shall be indicated on the label [12]. False declarations of botanical and geographical origins, together with sugar adulteration based on extraneous sugar addition, are the main identified non-conformities in the honey sector [13,14]. Correlation between food element composition and botanical and/or geographical origin was used in contemporary research for the purpose of using these correlations for traceability and as guarantees for consumers [15,16,17,18,19,20,21].

Tunisian beekeeping does not have a professional organization but is characterized by traditional practices that are still in an expansion phase. The most common bee is a native bee from north Africa, the *Apis mellifera intermissa*. This species is highly reproductive and very robust and active; it has adapted well to cold and dry climates. The honey consumption is mainly local, and its destination is the culinary field for the preparation of traditional sweets such as makroud, samsa and droo. Although in Tunisia honey has always been valued in traditional medicine, there are few investigations regarding some precious Tunisian varieties uncommon elsewhere, i.e., mint, thyme, rosemary or horehound [22].

In this line, the main objective of this research was to evaluate the element content in 36 honey samples of different botanical (wildflower, eucalyptus, eucalyptus red flowers, prickly pears, lemon-blossom, thyme, almond, rosemary and jujube) honeys and geographical regions (Sidi Bouzid, Nabeul and Sfax, Tunisia) inductively coupled plasma mass spectrometry (ICP-MS) and to use the multielement analysis together with chemometric tools to verify the botanical and the geographical origin of honeys.

## 2. Materials and Methods

### 2.1. Chemicals and Standard Solutions

Ultrapure water at a resistivity of 10 MΩ cm (J.T. Baker, Milan, Italy) and 65% concentrated nitric acid, trace metal analysis grade (J.T. Baker, Milan, Italy), were used in the study for all samples, blanks and standards. Hydrogen peroxide (30%) (J.T. Baker, Milan, Italy) was employed together with nitric acid for digestion samples.

Single element stock standard solutions of Fe, Zn, Cr, Ni, Cu, Se, Pb, V, Sb and As at a concentration of 1000 mg⋅L^−1^ in 2% nitric acid (Fluka, Milan, Italy) and of K, Ca, Mg, Na, Co, Ti, Mn, Hg and Cd at a concentration of 1000 mg⋅L^−1^ in 2% nitric acid (Merck, Darmstadt, Germany) were used to prepare multielement stock standard solutions at the concentration of 100 mg⋅L^−1^ for each element, which was used for calibration solutions and method validation. Five-point calibration curves were generated with the concentration in ranges between 0.5 and 20 µg⋅L^−1^ for Se, Pb, Ni, Cr, V, Sb, As, Cd and Hg; between 10 and 200 µg⋅L^−1^ for Fe, Zn, Ti, Mn, Cu and Co; and between 0.1 and 5 mg⋅L^−1^ for K, Ca, Na and Mg. A stock standard solution of Re at 1000 mg⋅L^−1^ in 2% nitric acid (Fluka, Milan, Italy) was used for the preparation of an internal standard at 0.5 mg⋅L^−1^in order to verify the sample digestion and to correct the volumetric changes. A stock standard solution of ^45^Sc, ^73^Ge, ^115^In and ^209^Bi at 1000 mg⋅L^−1^ in 2% nitric acid (Fluka, Milan, Italy) was used for the preparation of an on-line internal standard solution at level of 1.5 mg⋅L^−1^, in order to correct matrix deviation and instrumental drift.

The instrument performance was optimized using the ICP-MS solution containing 1 μg⋅L^−1^ of ^7^Li, ^59^Co, ^80^Y and ^205^Tl in 2% nitric acid (Agilent, Santa Clara, CA, USA). A solution of 5% sucrose served as a blank sample, and spiked samples used in the validation studies were also primed. Argon (N 5.0) and helium (N 5.5) were supplied by Rivoira gases (Milan, Italy).

### 2.2. Sample Collection

**Figure 1:**
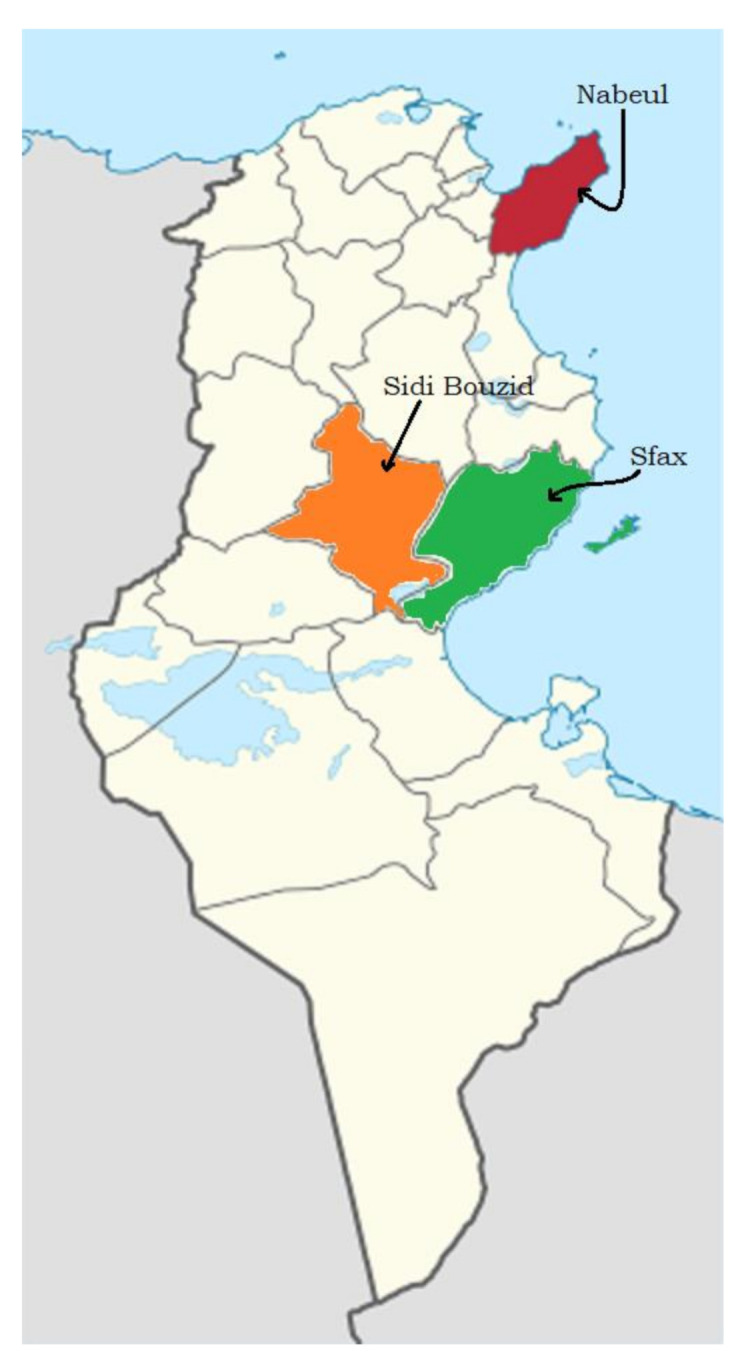
Samples area collection.

A total of 36 samples of honey were directly obtained from beekeepers in three districts of Tunisia in autumn 2019 (Figure 1). Twelve samples were from Sidi Bouzid governorate: 3 of wildflowers (W-SB), 3 of eucalyptus (E-SB), 3 of eucalyptus red flowers (ERF-SB) and 3 of prickly pears (PP-SB). Twelve samples were from Nabeul governorate: 4 of lemon-blossom (LB-N), 4 of thyme (T-N) and 4 of wildflowers (W-N). Twelve samples were from the Sfax governorate: 4 of almond (A-S), 4 of rosemary (R-S) and 4 of jujube (J-S). The honeys (about 125 g in weight) were collected in a clean glass jar, transported to the laboratory and were stored in the dark at 4 °C until analysis.

### 2.3. Sample Preparation

Samples were prepared according to Di Bella et al. [16]. After homogenization by heating at 40 °C, about 0.5 g of each samples was weighed, then we added 1 mL of Re standard solution at 0.5 mg⋅L^−1^, and the samples were digested with 7 mL of HNO_3_ (65%, *v*/*v*) and 1 mL of H_2_O_2_ (30%, *v*/*v*) in a microwave ETHOS 1 digestion system (Milestone, Bergamo, Italy) using the following instrumental parameters and setting: 15 min at 1000 W up to 200 °C, 15 min at 1000 W at 200 °C. After cooling, the digested sample solutions were quantitatively transferred into 25 mL Erlenmeyer flask and topped to the mark with ultrapure water. In order to analyze the potassium, calcium, sodium and magnesium contents, 1 mL of that solution was diluted with ultrapure water ten times. Blank and spiked samples were treated in the same way. All samples were processed in triplicate.

### 2.4. ICP-MS Analysis

Element concentrations were acquired using an Agilent 7500 cx (Agilent Technologies, Santa Clara, CA) ICP-MS spectrometer equipped with a 27.12 MHz radiofrequency solid-state generator at 1500 W, a MicroMist glass concentric pneumatic nebulizer and a cooled quartz Scott double pass type spray chamber. The instrument was also provided with an Octopole Reaction System (ORS) cell with helium gas, off-axis ion lens, quadrupole mass analyzer, electron multiplier detector, autosampler ASX520 (Cetac Technologies Inc., Omaha, NE, USA) and an integrated sample introduction system. ICP-MS analysis was carried out according the method reported in the previous published paper [21] and summarized in Table 1. All samples were analyzed in batches, with blank samples and known standards.

### 2.5. Method Validation Procedure

The EURACHEM criteria [23] were followed for the validation procedures. The developed method was validated for linearity of the calibration curves, sensibility, recovery, precision and repeatability. The standard solutions prepared at five concentrations for each analyte were analyzed by ICP-MS with checking the linearity between the concentrations and signal intensities. Each level of concentration was analyzed six times. The linearity of the calibration curves was evaluated by the respective correlation coefficients (*R*^2^). Sensitivity was assessed by establishing an instrumental detection limit (IDL) and an instrumental quantification limit (IQL) for each of the analytes. These values were determined as 3.3 σ/S and 10 σ/S, respectively, where σ represents the standard deviation of the response of the ten blanks and S is the slope of the calibration curve. Once instrumental IDLs and IQLs were calculated, the method detection limit (MDL) and method quantification limit (MQL) for the whole analytical procedure were determined by accounting for the sample preparation step. The recovery tests were performed using three replicates of spiking honey samples a three different concentration levels. The spiking levels used for recovery test were at 0.1, 0.2 and 0.5 mg⋅Kg^−1^ for Se, Pb, Ni, Cr, V, Sb, As, Cd and Hg; 0.5, 2 and 5 mg⋅Kg^−1^ for Fe, Zn, Ti, Mn, Cu and Co; and 0.5, 1 and 2 g⋅Kg^−1^ for K, Ca, Na and Mg. Each recovery was calculated as the percent between the value found with the calibration curve and the expected value derived by the added standard amount at the samples previously analyzed. The results are reported as average recovery values (%) among the three different concentration levels. The method precision was studied as repeatability and intermediate precision. The repeatability was determined by measuring the response for the three spiking honey samples four times each on the same days and one time each on twenty separate days.

### 2.6. Estimation of Dietary Intake

The estimation of the dietary intake of elements through the consumption of honey from the three districts of Tunisia points to the evaluation of the products’ quality and the possible health risks to consumers.

The daily intakes (DIs) were calculated by the equation DI (mg/day or µg/day) = C (mg/g or µg/g) × I (g), where C is the mean element concentration in analyzed samples and I is the adult daily intake for honey (1.8 and 0.3 g/capita/day for Europe and North Africa population, respectively) [24]. The calculated DIs were compared with the dietary reference values of essential elements reported by Communities Directive 2008/100/CE and EFSA [25,26].

The estimated daily intakes (EDIs) were calculated by the equation EDI (µg/kg_b.w._/day) = DI (µg/day)/Kg_b.w_, where the average body weight (b.w.) used was 70 kg. The calculated EDIs were compared with the international safety reference value for trace elements established by the government agency [27,28,29,30,31,32,33,34,35,36,37,38].

Additionally, a case study was constructed based on how many people really consume more honey than reported. In order to estimate how much honey can be consumed by a habitual consumer, we selected 25 healthy individuals, male and female, aged between 18 and 30 years, who eat substantial amounts of honey daily. We provided each participant with a 500 g bowl of honey. Participants were asked to consume this honey as usual for 2 weeks. Usually, 40% of the participants eat honey only for breakfast as a sweetener or spread on rusks, whereas the others consumed the honey also during the rest of the day. The honey consumption for each consumer was calculated by the difference between 500 g and the honey residue in the bowl.

### 2.7. Statistical Analysis

Data were analyzed statistically using the SPSS 13.0 software package for Windows (SPSS Inc., Chicago, IL, USA). Cobalt and Hg were suppressed from the multivariate analysis because they were < MQL in the total analyzed samples. For Cd, whenever the residue value was < MQL (in 17 % of the cases), the MDL/2 value was used. All concentrations were log-transformed to reduce the effect of outliers on skewing the data distribution and to bring the concentrations of the element within the same range. The Kruskal–Wallis test was performed to evaluate alpha diversities among the groups, and a significance level of *p* < 0.05 was adopted. To check for the classification among the honey’s botanical and geographical origins based on element content, principal component analysis (PCA) was applied. Prior to PCA, the variables were also autoscaled to provide equal importance because variables were measured at different concentration scales.

## 3. Results and Discussion

### 3.1. Method Validation

Table 2 summarizes the results of the method validation. For all elements under evaluation, *R*^2^ values were higher or equal to 0.9996. MDL values ranged from 0.005 to 6.135 µg⋅Kg^−1^, while MQL values ranged from 0.015 to 19.405 µg⋅Kg^−1^. The lowest average recovery was observed for selenium with 84.3%, whereas the highest was obtained for iron with 99.9%. Repeatability and intermediate precision were expressed as RSD% of the measurement made and were within 8 % and 11%, respectively. Based on these results, the method can be used in this study.

### 3.2. Results

The element concentrations (g·Kg^−1^ or mg·Kg^−1^ or µg·Kg^−1^) of the honey samples from different geographical areas of Tunisia are present in Table 3. In all cases, the most abundant elements were K (having mean values of 2.206 g·Kg^−1^), Na (0.780 g·Kg^−1^), Mg (0.665 g·Kg^−1^) and Ca (0.099 g·Kg^−1^), followed by Fe (5.130 mg·Kg^−1^) and Zn (2.212 mg·Kg^−1^). A total of 9 elements (Ti, Mn, Cu, Se, Pb, Ni, Cr, V and Sb) having mean values between 0.05 mg·Kg^−1^ and 1 mg·Kg^−1^ were trace elements. As mean values were 5.869 µg·Kg^−1^, and Cd mean values was 16.238 µg·Kg^−1^, but Cd was < LOQ in eucalyptus and prickly pear honey samples from Sidi Bouzid. The elements with a concentration < LOQ in all samples were Co and Hg. All results were in the range of the published data [3,7,10,11,13,16,18,22,39]. Significant differences in Ca, Ti, Mn, Cr, V, Sb, As and Cd content among honey botanical types were found (*p* < 0.05). Pairwise comparisons showed that jujube honeys had a higher mean value of Ca than other honeys; prickly pear and thyme honeys had a higher mean value of Ti than jujube and eucalyptus; jujube and rosemary honeys had a higher mean value of V than wildflower and thyme.

The Commission Regulation (EU) 2015/1005 of 25 June 2015 amending Regulation (EC) No 1881/2006 fixed the maximum permissible level for lead in honey at 0.1 mg·Kg^−1^. This new limit was fixed because lead is spread in the natural environment due to human activity, and honey is often subjected to contamination. Pb content exceeded this new upper limit in 69% of the total samples. This highlights that establishing the honey’s geographical origin may effectively assist in the establishment of health safety standards.

### 3.3. Principal Component Analysis 

**Figure 2:**
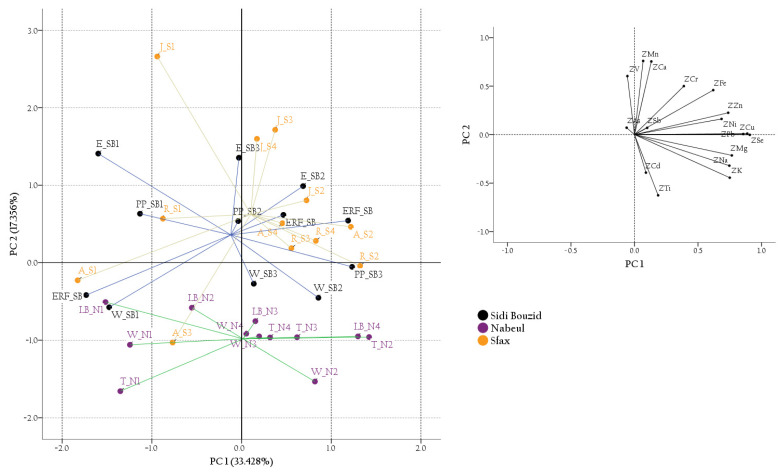
Two-dimensional score plots for the 36 honey samples categorized by geographical origin. Inserts: loadings plot for first principal component (PC1) and second principal component (PC2). Outcome obtained with 17 variables.

An exploratory analysis was performed using PCA on a matrix containing 36 rows (samples) and 17 columns (element concentrations). The correlation matrix was suitable for PCA (Kaiser-Meyer-Olkin values of 0.671; Bartlett’s Test of Sphericity values equal to 394.251, with *p*-value < 0.0005) and shows 56% of the coefficients with values significantly higher than 95%. Four principal components with eigenvalues exceeding one (5.683, 2.951, 2.169 and 1.671) were extracted according to the Kaiser criterion. The extracted components explained up to 73.376% of total variance (33.428%, 17.356%, 12.760% and 9.831%, respectively). The distribution of samples in the space of a score plot showed a large amount of overlap among samples of different groups according to both geographical and botanical origin (Figure 2).

Taking into account only those variables that showed significant influence on the first PCA and that matched with the results of the Kruskal–Wallis test, and performing varimax rotation, new, an improved PCA was obtained. The correlation matrix factorability was checked and achieved again (KMO values of 0.605; Bartlett’s Test of Sphericity values equal to 66.328, with *p*-value < 0.0005) and show 47% of the coefficient with values with significances higher than 95%. The highest positive correlations were observed for V-Mn (0.626) and V-Cr (0.466), while the highest negative correlations were observed for Ca-Ti (−0.630) and Cs-Cd (−0.456). Two principal components with eigenvalues exceeding one (2.417 and 1.509) were extracted, and the percentages of the total variance in the rotated model were 33.686% and 31.754%, respectively. V, Cr and Mn were correlated to the first component, all with positive values (0.854, 0.807 and 0.766, respectively). The second component showed the highest positive correlation with Cd (0.704) and Ti (0.703), while a negative correlation was be observed for Ca (−0.884).

**Figure 3:**
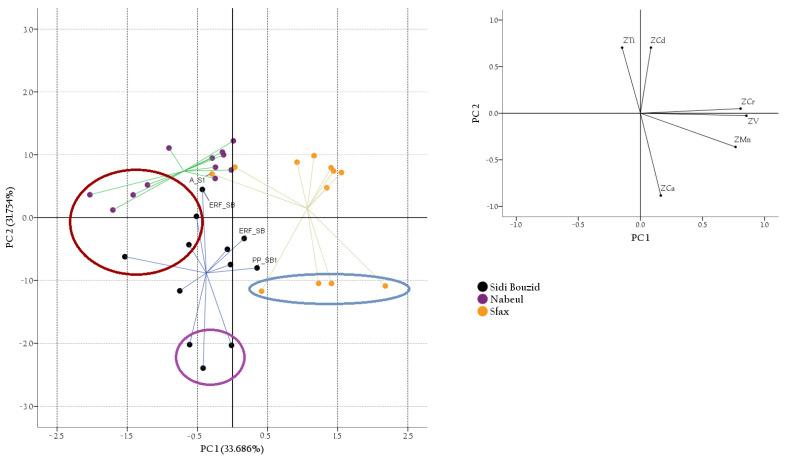
Two-dimensional score plots for the 36 honey samples categorized by geographical origin. Inserts: loadings plot for PC1 and PC2. Outcome obtained with 6 variables and with Varimax rotation.

Figure 3 shows the PC1/PC2 score plot where three clusters are distinguishable according to the geographic collection area. PC2 axis split samples between Sfax and the others obtained from Sidi Bouzid and Nabeul. The first ones showed almost always positive PC1 scores while the remaining showed almost always negative PC1 ones. Moreover, samples from Sidi Bouzid were separated from the samples from Nabeul by PC1, with the honeys obtained from Nabeul at positive PC2 scores, while the others are at negative PC2 scores. Thus, the Sfax samples showed the highest Cr, V and Mn concentrations; the samples collected from Nabeul had the highest concentrations of Ti, and the lowest Mn and Ca concentrations.

The samples from Sidi Bouzid had the lowest Cd residues. It can also be stressed that the only sample with negative PC1 scores among the honey from Sfax was a sample of almond that showed a lower Mn and Cr concentration than the other honey from its cluster. Among the honeys from Sidi Bouzid, only three samples were not positioned in the third quadrant. It is also possible to verify that the groups of jujube, eucalyptus and wildflower honey form separable clusters. Therefore, in this study, the correlation between the honey and the botanical and geographical area on the basis of mineral elements was in some measure achieved.

### 3.4. Elements Uptake by Honeys

The honeys’ quality and the possible health risks to consumers were evaluated by calculation of daily intakes (DIs) and estimated daily intakes (EDIs) as reported in Section 2.6. The calculate DIs were compared with the dietary reference values for elements considered essential for human nutrition [25,26], whereas the calculate EDIs were compared with the international safety reference value for trace elements considered toxic or potentially toxic [27,28,29,30,31,32,33,34,35,36,37,38,40]. Shares of dietary reference values of essential elements and of international safety reference values for not essential elements by consumption of the honey from the three districts of Tunisia analyzed in this study are reported in Table 4 and Table 5.

Mean exposure values satisfy a very low percentage of the reference value for all elements. This small exposure is related to the small amount consumed in common diets (1.8 and 0.3 g/capita/day for Europe and North Africa population, respectively) [24]. Many people really consume more honey than reported. According to results of our case study, the estimated amount of honey consumed by a habitual honey consumer was of 15 g/capita/day. This honey daily intake was used to recalculate the percentage of international safety reference values for lead, because this element exceeded the maximum permissible level fixed by Regulation (EC) No 1881/2006 (0.1 mg·Kg^−1^) in 69 % of the total samples. Lead uptake reaches the maximum values in the honey from Sfax (11.4%), followed by honey from Nabeul (9.0%) and from Sidi Bouzid (8.2%). Even though mean exposure values are well below the protection limits for this element, for a habitual honey consumer, if we have other contaminated food items in the principal diet, exposure will easily exceed the limits.

## 4. Conclusions

Honeys from different provinces of the Tunisia were characterized according to their element composition. The results were used to discriminate the botanical and geographical origin of honey samples applying multivariate data analysis. The PCA model was able to distinguish three clusters according to the geographic collection area if Mn, Ca, V, Cr, Ti and Cd were used as variables. It is also possible to verify that jujube, eucalyptus and wildflower honeys were separable as clusters. In compliance with the results of the present study, the intake of elements was small but in line with aliments such honey. Concerning lead, although the amount in analyzed samples was higher than the legal limit, the estimated daily intake showed that health risks are, however, small.

## Figures and Tables

**Table 1:** Instrument operating parameters for inductively coupled plasma mass spectrometry (ICP-MS) analyses.

RF Power	1550 W
Plasma/auxiliary/carrier gas flow rate	14/0.8/0.93 L⋅min^−1^
Helium collision gas flow rate	4.5 mL⋅min^−1^
Spray chamber temperature	2.7 °C
Sample depth	9 mm
Sample introduction flow rate	1 mL⋅min^−1^
Nebulizer pump	0.1rps
Extract lens 1	1.5 V
Octopole collision system setting	He mode for Na, Mg, K, Ca, Ti, V, Cr, Fe, Co, Ni, Cu, As, Se; No-gas mode for Mn, Zn, Cd, Sb, Hg, and Pb
Monitored isotopes	^23^Na, ^24^Mg, ^39^K, ^44^Ca, ^47^Ti, ^51^V, ^52^Cr, ^55^Mn, ^56^Fe, ^59^Co, ^60^Ni, ^63^Cu, ^66^Zn, ^75^As, ^78^Se, ^111^Cd, ^121^Sb, ^202^Hg and ^208^Pb
On-line internal standards	^45^Sc for Na, Mg, K, Ca, Ti, V, Cr, Mn, Fe and Co; ^72^Ge for Ni, Cu, Zn, As and Se; ^103^Rh for Cd and Sb; ^209^Bi for Hg and Pb
Integration times	0.8 s/point for Se; 0.5 s/point for As; 0.2 s/point for Cu, Cr and Ni; 0.1 s/point for the other elements
Point for mass	3 (3 replicates acquisitions)

**Table 2:** Method linearity, method detection limits (MDLs), method quantification limits (MQLs), recovery, repeatability and intermediate precision.

Element	R^2^	MDLs (µg·Kg^−1^)	MQLs (µg·Kg^−1^)	Recovery (%)	Repeatability (RSD%)	Intermediate Precision (RSD%)
K	0.9996	1.055	3.165	92.3 ± 1.6	3.2	5.0
Ca	0.9998	4.605	12.815	97.2 ± 2.9	5.4	10
Na	0.9998	6.135	19.405	90.3 ± 1.4	1.7	2.5
Mg	0.9996	1.840	5.520	95.1 ± 3.6	1.5	2.6
Fe	0.9997	0.700	2.100	99.9 ± 0.8	2.1	3.6
Zn	0.9996	2.825	8.475	97.9 ± 1.5	4.0	5.2
Ti	0.9997	0.065	0.195	90.4 ± 1.2	2.6	4.6
Mn	0.9998	0.035	0.105	98.2 ± 1.0	1.4	2.3
Cu	0.9996	0.745	2.235	97.7 ± 0.7	8.0	11
Co	0.9998	0.010	0.030	91.3 ± 2.7	2.2	4.9
Se	0.9996	3.105	9.315	84.3 ± 3.9	3.9	8
Pb	0.9998	0.010	0.030	90.8 ± 2.6	2.4	4.6
Ni	0.9998	0.880	2.640	93.5 ± 1.2	1.1	2.8
Cr	0.9997	0.025	0.075	78.0 ± 2.3	4.9	9.7
V	0.9998	0.065	0.195	97.0 ± 2.8	2.6	4.5
Sb	0.9998	0.020	0.060	97.8 ± 1.3	3.4	4.4
As	0.9997	0.005	0.015	97.6 ± 3.6	1.0	2.6
Cd	0.9997	0.010	0.030	99.5 ± 1.2	6.1	7.5
Hg	0.9998	0.010	0.030	98.3 ± 1.0	1.5	2.3

**Table 3:** Mineral contents (mean ± standard deviation) for Tunisian honey samples and results of Kruskal–Wallis test for honey samples categorized by their botanical and geographical origin.

	K (g·kg^−1^)	Ca (g·kg^−1^)	Na (g·kg^−1^)	Mg (g·kg^−1^)	Fe (mg·kg^−1^)	Zn (mg·kg^−1^)	Ti (mg·kg^−1^)	Mn (mg·kg^−1^)	Cu (mg·kg^−1^)	Se (mg·kg^−1^)	Pb (mg·kg^−1^)	Ni (mg·kg^−1^)	Cr (mg·kg^−1^)	V (mg·kg^−1^)	Sb (mg·kg^−1^)	As (µg·kg^−1^)	Cd (µg·kg^−1^)
Botanical Origin																	
Wildflower	2.20 ± 0.62	0.07 ± 0.01 C D	0.61 ± 0.39	0.71 ± 0.51	3.25 ± 1.53	2.40 ± 1.13	0.93 ± 0.26 A C	0.31 ± 0.11 A	0.66 ± 0.31	0.15 ± 0.13	0.24 ± 0.12	0.38 ± 0.21	0.14 ± 0.14 A	0.03 ± 0.01 A	0.09 ± 0.01 A	3.16 ± 1.40 A	19.01 ± 4.66 B C
Eucalyptus	1.58 ± 0.72	0.17 ± 0.02 D	0.56 ± 0.44	0.42 ± 0.07	7.10 ± 3.28	2.06 ± 0.48	0.61 ± 0.06 A	1.25 ± 0.09 B	0.80 ± 0.45	0.13 ± 0.11	0.25 ± 0.20	0.22 ± 0.15	0.13 ± 0.03 B	0.05 ± 0.01 A B	0.10 ± 0.02 A B	19.08 ± 7.82 B	n.d.
Euc. red flowers	1.52 ± 0.47	0.11 ± 0.07 D	0.80 ± 0.47	0.37 ± 0.21	7.15 ± 4.67	2.61 ± 0.82	0.80 ± 0.05 A C	0.58 ± 0.31 A B C	1.26 ± 1.01	0.21 ± 0.18	0.17 ± 0.16	0.42 ± 0.35	0.18 ± 0.01 A B	0.05 ± 0.01 A B	0.08 ± 0.01 A	9.22 ± 5.29 B C	27.71 ± 12.08 B
Prickly pears	2.53 ± 1.74	0.14 ± 0.04D	1.03 ± 0.97	0.93 ± 0.81	5.88 ± 2.39	2.01 ± 0.71	1.35 ± 0.11 B	0.91 ± 0.44 B	0.73 ± 0.38	0.21 ± 0.17	0.18 ± 0.18	0.23 ± 0.11	0.15 ± 0.02 A B	0.06 ± 0.01 B C	0.11 ± 0.01 B	3.55 ± 0.52 A B	n.d.
Lemon-blossom	2.62 ± 1.21	0.05 ± 0.01 A C	1.22 ± 0.95	0.84 ± 0.64	3.85 ± 1.31	1.69 ± 0.56	1.17 ± 0.05 B C	0.44 ± 0.08 C	0.86 ± 0.33	0.14 ± 0.23	0.13 ± 0.15	0.32 ± 0.19	0.21 ± 0.06 A B	0.05 ± 0.01 A C	0.08 ± 0.01 A	12.16 ± 8.88 B C	17.72 ± 1.26 A B
Thyme	3.19 ± 1.29	0.03 ± 0.01 A	1.40 ± 0.70	1.20 ± 0.55	5.17 ± 3.19	1.74 ± 0.79	1.31 ± 0.15 B	0.44 ± 0.17 C	0.84 ± 0.44	0.26 ± 0.18	0.19 ± 0.16	0.22 ± 0.09	0.17 ± 0.06 A B	0.04 ± 0.00 A	0.11 ± 0.01 B	2.65 ± 0.60 A	21.99 ± 8.87 B C
Almond	1.53 ± 1.26	0.04 ± 0.01 A C	0.80 ± 0.42	0.78 ± 0.45	2.70 ± 1.87	2.13 ± 1.29	1.06 ± 0.05 C	1.49 ± 0.98 B	0.53 ± 0.21	0.19 ± 0.16	0.19 ± 0.14	0.51 ± 0.35	0.19 ± 0.11 A B	0.06 ± 0.01 B C	0.13 ± 0.04 B	2.66 ± 1.23 A	19.20 ± 2.93 B C
Rosemary	3.11 ± 1.61	0.04 ± 0.01 A C	0.84 ± 0.52	1.01 ± 0.17	7.97 ± 3.96	2.81 ± 0.61	1.08 ± 0.05 B C	1.02 ± 0.53 B	0.81 ± 0.32	0.19 ± 0.15	0.27 ± 0.17	0.20 ± 0.07	0.35 ± 0.04 B	0.07 ± 0.01 B	0.12 ± 0.02 B	3.39 ± 1.68 A C	16.40 ± 2.48 A C
Jujube	1.32 ± 1.09	0.29 ± 0.06 B	0.57 ± 0.35	0.49 ± 0.20	5.70 ± 1.80	2.34 ± 0.19	0.59 ± 0.05 A	1.06 ± 0.36 B	0.76 ± 0.38	0.24 ± 0.18	0.34 ± 0.22	0.45 ± 0.17	0.50 ± 0.28 B	0.07 ± 0.01 B	0.09 ± 0.01 A	2.55 ± 1.10 A	13.79 ± 5.04 A B
F statistic	11.609	29.666	6.742	9.987	10.189	6.731	27.669	23.176	4154	1.290	4.580	8.804	17.289	27.270	18.857	21.007	20.839
Significant level	0.198	**0.001**	0.565	0.266	0.252	0.566	**0.001**	**0.003**	0.843	0.996	0.801	0.359	**0.027**	**0.001**	**0.016**	**0.007**	**0.008**
Geographical origin																	
Sidi Bouzid	2.00 ± 1.03	0.13 ± 0.05 B	0.81 ± 0.56	0.59 ± 0.46	6.14 ± 2.98	2.07 ± 0.64	0.86 ± 0.31 B	0.78 ± 0.41 B	0.87 ± 0.58	0.16 ± 0.13	0.19 ± 0.15	0.32 ± 0.23	0.17 ± 0.09 B	0.04 ± 0.01 A	0.09 ± 0.02	8.48 ± 8.06	11.90 ± 12.50
Nabeul	2.62 ± 1.07	0.05 ± 0.01 A	1.02 ± 0.77	0.94 ± 0.65	3.79 ± 2.17	2.14 ± 1.02	1.20 ± 0.13 A	0.37 ± 0.15 A	0.78 ± 0.34	0.19 ± 0.18	0.21 ± 0.14	0.30 ± 0.15	0.15 ± 0.07 B	0.04 ± 0.01 A	0.10 ± 0.01	6.26 ± 6.43	20.36 ± 5.39
Sfax	1.99 ± 1.47	0.12 ± 0.13 A	0.74 ± 0.42	0.76 ± 0.35	5.46 ± 3.35	2.43 ± 0.81	0.91 ± 0.24 B	1.19 ± 0.65 B	0.70 ± 0.31	0.21 ± 0.15	0.27 ± 0.17	0.38 ± 0.25	0.35 ± 0.21 A	0.07 ± 0.01 B	0.11 ± 0.03	2.87 ± 1.29	16.46 ± 4.03
F statistic	3.066	9.394	0.464	2.477	3.968	1.227	11.722	16.221	0.284	0.224	1.236	0.407	8.565	19.352	3.246	5.265	6.224
Sign. level	0.216	**0.009**	0.793	0.290	0.138	0.542	**0.003**	**0.001**	0.868	0.894	0.539	0.816	**0.014**	**0.001**	0.197	0.072	0.051

Bold values showed significant levels at *p* < 0.05; A, B, C and D indicate homogeneous groups at α = 0.05: honey types which do not differ from each other are designated by same letter. Significant differences among honeys from different geographical origin were for Ca, Ti, Mn, Cr and V (*p* < 0.05). Honeys from Nabeul had the lowest Ca and Mn content and the highest Ti content, whereas honeys from Sfax had the highest Cr and V content.

**Table 4:** Shares of dietary reference values of essential elements by consumption of honey from the three districts of Tunisia.

		DI (mg/d or µg/d)			RDA [25] (mg/d or µg/d)	AI [26] (mg/d or µg/d)	AR [26] (mg/d or µg/d)	PRI [26] (mg/d or µg/d)	UL [26] (mg/d or µg/d)	% of RDA or AI	% of UL
		Sidi Bouzid	Nabeul	Sfax							
K											
	Europe	3.61	**4.72**	3.58	**2000**	3500				0.24%	
	North Africa	0.60	**0.79**	0.60						0.04%	
Mg											
	Europe	1.06	**1.69**	1.37	**375**	350			**250**	0.45%	0.68%
	North Africa	0.18	**0.28**	0.23						0.08%	0.08%
Ca											
	Europe	**0.23**	0.09	0.22	**800**		860	1000	**2500**	0.03%	0.01%
	North Africa	**0.04**	0.01	0.04						0.00%	0.00%
Na											
	Europe	1.46	**1.84**	1.33		**2000**				0.09%	
	North Africa	0.24	**0.31**	0.22						0.02%	
Fe											
	Europe	**1 × 10^−2^**	7 × 10^−3^	1 × 10^−2^	**14**		6	11		0.08%	
	North Africa	**2 × 10^−3^**	1 × 10^−3^	2 × 10^−3^						0.01%	
Mn											
	Europe	1 × 10^−3^	7 × 10^−4^	**2 × 10^−3^**	**2**	3				0.11%	
	North Africa	2 × 10^−4^	1 × 10^−4^	**4 × 10^−4^**						0.02%	
Zn											
	Europe	4 × 10^−3^	4 × 10^−3^	**4 × 10^−3^**	**10**		7.5–9.3–11–12.7 *	9.4–11.7–14–16.3 *	**25**	0.04%	0.02%
	North Africa	6 × 10^−4^	6 × 10^−4^	**7 × 10^−4^**						0.01%	0.00%
Cu											
	Europe	**2 × 10^−3^**	1 × 10^−3^	1 × 10^−3^	**1**	1.6			5	0.16%	0.03%
	North Africa	**3 × 10^−4^**	2 × 10^−4^	2 × 10^−4^						0.03%	0.01%
Cr											
	Europe	*0.30*	*0.28*	***0.62***	***40***					0.76%	
	North Africa	*0.05*	*0.05*	***0.10***						0.13%	
Se											
	Europe	*0.29*	*0.35*	***0.37***	***55***	*70*			***300***	0.68%	0.12%
	North Africa	*0.05*	*0.06*	***0.06***						0.11%	0.02%

Data for % calculation reported in bold; * ARs and PRIs for zinc are provided for four levels of phytate intake (LPI): 300, 600, 900 and 1200 mg/day. Abbreviations: DI, Dietary Intake; RDA, Recommended Dietary Allowances; AI, Adequate Intakes; AR, Average Requirement; PRI, Population Reference Intake; UL, Tolerable Upper Intake Level.

**Table 5:** Shares of international safety reference values for trace elements by consumption of honey from the three districts of Tunisia.

		EDI (µg/Kg_b.w._/d)			TDI (µg/Kg_b.w._/d)	TWI (µg/Kg_b.w._/w)	BMDL01 (µg/Kg_b.w._/d)	PTWI (µg/Kg_b.w._/w)	UI (µg/Kg_b.w._/w)	PMTDI (µg/Kg_b.w._/d)	% of TDI or TWI or BMDL01 or PTWI	% of UI or PMTDI
		Sidi Bouzid	Nabeul	Sfax								
Ti												
	Europe	2 × 10^−2^	3 × 10^−2^	2 × 10^−2^	--------------------------------------------- not delivered ------------------------------------------------------------							
	North Africa	4 × 10^−3^	5 × 10^−3^	4 × 10^−3^								
Co												
	Europe	n.d.	n.d.	n.d.					1.6 [32]			
	North Africa	n.d.	n.d.	n.d.								
Sb												
	Europe	2 × 10^−3^	2 × 10^−3^	**3 × 10^−3^**					**6** [27]			0.34%
	North Africa	4 × 10^−4^	4 × 10^−4^	**5 × 10^−4^**								0.06%
V												
	Europe	1 × 10^−3^	1 × 10^−3^	2 × 10^−3^	--------------------------------------------- not delivered ------------------------------------------------------------							
	North Africa	2 × 10^−4^	2 × 10^−4^	3 × 10^−4^								
Ni												
	Europe	8 × 10^−3^	8 × 10^−3^	**1 × 10^−2^**	**22** [38]				**2.8** [33]		0.04%	2.47%
	North Africa	1 × 10^−3^	1 × 10^−3^	**2 × 10^−3^**							0.01%	0.41%
Pb												
	Europe	5 × 10^−3^	5 × 10^−3^	**7 × 10^−3^**			**0.5** [29]	25 [36]			1.37%	
	North Africa	8 × 10^−4^	9 × 10^−4^	**1 × 10^−3^**							0.23%	
As												
	Europe	**2 × 10^−4^**	2 × 10^−4^	**7 × 10^−5^**			**0.3**-8 [28]	15 [35]			0.07%	
	North Africa	**4 × 10^−5^**	3 × 10^−5^	**1 × 10^−5^**							0.01%	
Cd												
	Europe	3 × 10^−4^	**5 × 10^−4^**	4 × 10^−4^		**2.5** [30]		**7** [37]			0.15%	
	North Africa	5 × 10^−5^	9 × 10^−5^	7 × 10^−5^							0.02%	
Hg												
	Europe	n.d.	n.d.	n.d.		4 [31]		5 [34]				
	North Africa	n.d.	n.d.	n.d.								
Se												
	Europe	4 × 10^−3^	5 × 10^−3^	**5 × 10^−3^**				**66** [40]		**9.4** [40]	0.06%	0.06%
	North Africa	7 × 10^−4^	8 × 10^−4^	**9 × 10^−4^**							0.01%	0.01%
Zn												
	Europe	5 × 10^−2^	6 × 10^−2^	**6 × 10^−2^**				**7000** [40]		**1000** [40]	0.01%	0.01%
	North Africa	9 × 10^−3^	9 × 10^−3^	**1 × 10^−2^**							0.001%	0.001%
Mn												
	Europe	2 × 10^−2^	1 × 10^−2^	**3 × 10^−2^**				**2500** [40]		**360** [40]	0.01%	0.01%
	North Africa	3 × 10^−3^	2 × 10^−3^	**5 × 10^−3^**							0.001%	0.001%
Cu												
	Europe	**2 × 10^−2^**	2 × 10^−2^	2 × 10^−2^				**3500** [40]		**500** [40]	0.001%	0.001%
	North Africa	**4 × 10^−3^**	3 × 10^−3^	3 × 10^−3^							0.001%	0.001%

Data for % calculation reported in bold. Abbreviations: EDI, Estimated Daily Intake; TDI, Tolerable Dietary Intake; TWI, Tolerable Weekly Intake; BMDL01, Benchmark Dose Lower Confidence Limit 01; PTWI, Provisional Tolerable Weekly Intake; UI, tolerable upper Intake level; PMTDI, Provisional Maximum Tolerable Daily Intake.
